# Mental Health during fatherhoood. Biopsychosocial aspects and questionnaire for depression PHQ9

**DOI:** 10.1192/j.eurpsy.2023.609

**Published:** 2023-07-19

**Authors:** M. O. Solis Correa, A. Alvarado Dafonte, F. Vilchez Español

**Affiliations:** ^1^Hospital Neurotraumatologico Jaén, Jaén; ^2^Hospital Antequera, Málaga; ^3^Hospital Neurotraumatológico, Jaén, Spain

## Abstract

**Introduction:**

Both women and men experience potentially stressful events during their reproductive periods and both are at risk of developing peripartum depression. Men have a reproductive period that is difficult to define, and research on their mental health has rarely considered the effects of paternity. A prevalence of postpartum depressive symptomatology of 10.4% has been described worldwide (Paulson J et al. 2010). Paternal depression is also a risk factor for peripartum maternal depression (Escribá et al, 2011; Paulson et al., 2016). Among the risk factors for developing postpartum depression in men are identified: personal history of depression, conflictive relationship, lack of family and social support, unemployment, older age, lower educational level, and the father’s ability to support his new role as a father (Morse et al., 2000).

**Objectives:**

Screening to investigate and identify early objective biomarkers in recent fathers of early depression.

**Methods:**

An anonymous survey is carried out through GoogleForms, to 57 men, fathers, with children born alive under 1 year of age, which includes biopsychosocial aspects and a questionnaire for depression: PHQ9.

**Results:**

Of the total of 57 parents, the average age is 36 years. 4 of them are unemployed, 1 is a student, the rest have active work or parent´s licency. Only 10% refer to present economic problems. 36% reported that their partner had a risky pregnancy and 22% had a peripartum complication. 9% describe an unsatisfactory or very unsatisfactory relationship with the mother of their child(ren). 51% have a personal and/or family history of depression and/or anxiety. 57% are overwhelmed in their role as fathers. 33% feel they have little or no social/emotional support. 5% have increased the consumption of alcohol/psychotropic medication and 94% report that their sleep pattern has been affected. 3.5% refer self-injurious thoughts or that they would be better off dead. 14% have considered requesting/consulting with a psychiatrist/psychologist since the arrival of the baby.

In relation to PHQ 9, 5% present moderate/severe depression.

**Conclusions:**

In conclusion, it seems relevant to think about a screening to investigate and identify early objective biomarkers and rapid intervention, not only in mothers but also in fathers and thus take a first step to broaden the view from the mother-child dyad to the triad, thus understanding that mental health does not exist in isolation, it is a contextual and relational phenomenon and also reduce the negative impact of this problem, such as: dysfunction and family well-being, marital satisfaction, growth and development of your child/ren . In this context, primary care health professionals (midwives and primary care doctors) could play a fundamental role in recognizing the importance of incorporating parents as relevant figures in health .

**Disclosure of Interest:**

None Declared

